# Changing Trends of Cutaneous Lupus Erythematosus (CLE) in a Tertiary Care Hospital in Northern Odisha, India

**DOI:** 10.7759/cureus.40466

**Published:** 2023-06-15

**Authors:** Debabrata Nayak, Binodini Behera, Sambit Ranjan Dalei, Amit Kumar Mishra, Paraini Marandi

**Affiliations:** 1 Dermatology, Pandit Raghunath Murmu Medical College, Baripada, IND; 2 Dermatology, Fakir Mohan Medical College, Balasore, IND; 3 Community and Family Medicine, All India Institute of Medical Sciences, Raipur, IND; 4 Dermatology, Bhima Bhoi Medical College, Balangir, IND

**Keywords:** india, baripada, northern odisha, cutaneous lupus erythematosus, changing trends

## Abstract

Background: Cutaneous lupus erythematosus (CLE) is a chronic autoimmune inflammatory skin disorder. Several studies have been published regarding its prevalence, demographic details, clinical spectrum, and various associated factors. In our out patient department (OPD), we noticed an increase in the number of cases of CLE in our area in the last few years. Therefore, the current cross-sectional study was conducted to assess the trends of CLE among patients who reported to a tertiary care hospital.

Materials and Methods: The current study is a record-based cross-sectional study of 81 patients of CLE, who attended the dermatology OPD of a tertiary care hospital. Data were collected from 2017 to 2022 and were divided into three different periods of time (2017-2018, 2019-2020, and 2021-2022). Demographic details, clinical examination findings, and laboratory investigation reports were also collected.

Results: There was a rising trend in the cases of CLE. Females outnumbered males (2:1, 66.67%). The increase in cases from 2017-2018 to 2019-2020 was 157% and from 2019-2020 to 2021-2022 was 204%. In 2021-2022, 52% of cases of chronic CLE (CCLE) were males. Photosensitivity was the most common finding. The majority of patients were addicted to smoking.

Conclusion: The current study noticed an increasing trend in all types of CLE. So, this rising trend should be investigated for possible triggering factors like climatic changes, infections, and drug factors with a larger sample size.

## Introduction

Lupus erythematosus (LE) is a chronic autoimmune inflammatory skin disorder with various presentations along its clinical spectrum. It can present solely as a mild cutaneous form or as a severe multisystem disorder [1]. Based on the histopathological features, Gilliam and Sontheimer classified the cutaneous manifestations of LE as specific, i.e. cutaneous LE (CLE) or non-specific on the basis of clinical, histological, and prognostic criteria. From a clinical and prognostic point of view, specific skin lesions are further categorized into three subtypes: acute CLE (ACLE), subacute CLE (SCLE), and chronic CLE (CCLE) [2]. ACLE, most frequently associated with systemic LE occurs more commonly in women in the second and third decades with either localized or generalized manifestations [3]. SCLE is characterized by eruptions over photosensitive areas like the neck, anterior chest, upper back, and shoulders. It is routinely associated with positive anti-Ro antibodies and may be induced by different types of drugs. Discoid LE is the most prevalent form of CCLE, demonstrated by indurated scaly plaques on the scalp, face, and external ears with features of scarring and pigmentary changes [4]. Although a few epidemiological studies have been conducted on the clinical profile of CLE, the data on the prevalence of cutaneous LE in northern Odisha, mostly having a tribal population still remains insufficient. Furthermore, there are no studies available to observe the recent changes in the demographic profile, prevalence, and clinical characteristics of patients. The aim of this study was to observe the changing trends of cutaneous LE patients who visited the dermatology department of a tertiary care hospital.

## Materials and methods

Detailed history and demographic data of patients visiting the Department of Dermatology and Venereology of Pandit Raghunath Murmu Medical College and Hospital, Baripada, a tertiary care hospital in Odisha were taken. Those details were always recorded in the available registers and files. The study was initiated after the due approval from the Institute Ethics Committee 7/6th IEC/PRMMCH. Eligible patients diagnosed clinically with cutaneous LE between January 2017 and December 2022 were identified from the available records and included in the study. Data were extracted from the available records using a case data extraction sheet. To study the change in trends, the patients were divided into three groups, i.e. patients’ details recorded for two consecutive years were taken as a single time unit; patients who presented to the OPD from January 2017 to December 2018 formed Group A; patients from January 2019 to December 2020 formed Group B; and patients from January 2021 to December 2022 constituted Group C. 

Study tools

There is a norm of proper history taking and clinical examination (general and systemic), dermatological examination, laboratory investigations such as complete blood count, liver function test, renal function test, erythrocyte sedimentation rate, routine and microscopic examination of urine and antinuclear antibody (ANA) testing in the Department of Dermatology and Venereology of Pandit Raghunath Murmu Medical College and Hospital, Baripada and the clinical examination/laboratory findings are recorded in the available records/files. Histopathological examination was done in doubtful cases. A data extraction sheet was developed and used to collect data regarding the patients as per the objective of the study. Detailed information of all patients regarding demography such as age, sex, area of residence, occupation, addiction history, past history of any photodermatitis, systemic complaints, any significant drug history, and family history of LE were extracted from the records.

Analysis of data

After the compilation of the required data, it was cleaned and analyzed with the IBM Statistical Package for the Social Sciences (SPSS) Statistics for Windows, Version 21.0 (Developed by IBM Corp., Armonk, New York). Descriptive data were presented with frequency and percentage.

## Results

Some 81 patients were enrolled in the study from 2017 to 2022. Some 14 patients of cutaneous LE presented to our OPD during 2017-2018 (Group A), whereas the number was 22 during 2019-2020 (Group B), and 45 during 2021-2022 (Group C). The demographic characteristics of all 81 patients attending the facility are shown in Table 1.

**Table 1:** Demographic details of patients (N=81). ACLE, acute CLE; SCLE, subacute CLE; CCLE, chronic CLE; CLE, cutaneous lupus erythematosus

Study period	2017-2018 (Group A)			2019-2020 (Group B)			2021-2022 (Group C)		
Cutaneous LE type	ACLE	SCLE	CCLE	ACLE	SCLE	CCLE	ACLE	SCLE	CCLE
No. of patients	3	2	9	5	3	14	11	7	27
Gender/Sex									
Male	1	1	2	1	1	3	2	2	14
Female	2	1	7	4	2	11	9	5	13
Age grouping in years									
11-20	1	0	0	1	0	0	3	1	1
21-30	1	1	1	3	2	2	5	2	2
31-40	1	1	1	1	1	2	2	3	6
41-50	0	0	4	0	0	6	1	1	11
>50	0	0	3	0	0	4	0	0	7
Duration (in months)									
	1.66 ± 1.15	3 ± 1.41	8.88 ± 3.4	2 ± 7.62	2.33 ± 0.58	7.42 ± 3	1.9 ± 0.94	3 ± 0.81	7.55 ± 3.84
Areawise distribution									
Rural	2	1	7	4	2	11	6	4	10
Urban	1	1	2	1	1	3	5	3	17
Occupation profile									
Business	0	0	0	0	0	0	0	0	1
Independent profession	1	0	1	0	1	2	3	3	2
Farmer	1	1	3	2	1	6	3	2	9
Laborer	0	1	3	2	0	4	2	2	11
None	1	0	2	1	1	2	3	0	4

**Figure 1:**
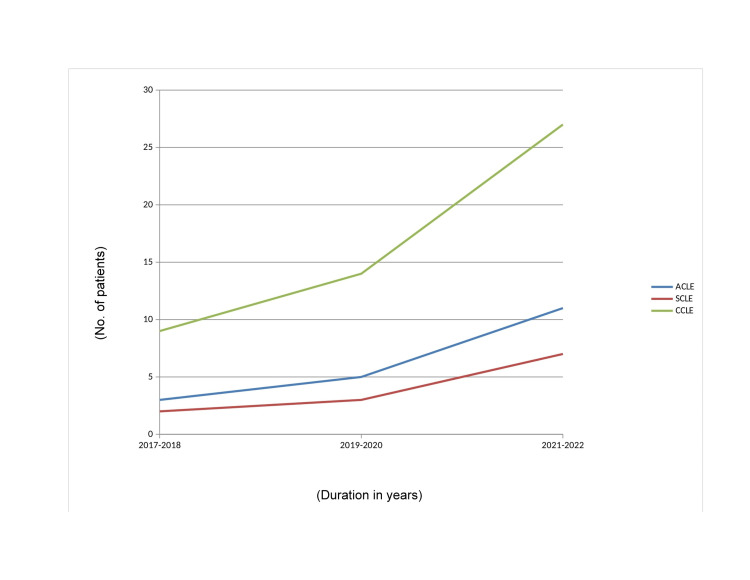
Changing trends in types of CLE -- this figure shows a gradual increase in cases of CLE over the years from 2017 to 2022. CLE, cutaneous lupus erythematosus

An increasing trend was observed among the CLE patients over the years ranging from 2017 to 2022. Throughout the study period, CCLE was the most common variant of cutaneous LE as shown in Figure 1.

**Figure 2:**
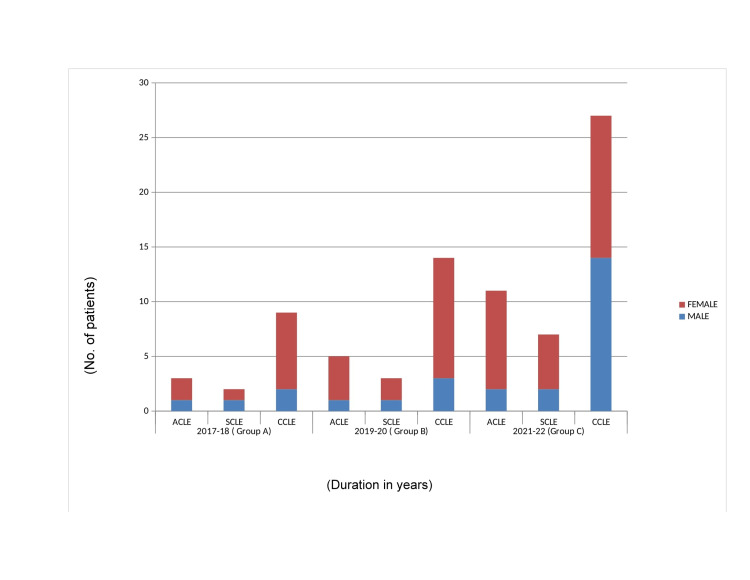
Sex distribution. The figure shows sex distribution among patients. The figure shows CLE is more common in females, but in 2021-2022 males with CCLE outnumber females. CCLE, chronic CLE; CLE, cutaneous lupus erythematosus

While examining our findings, CLE was observed in a greater number in women (10:4, 17:5, 27:18). But in Group C males suffering from CCLE outnumbered females (Figure 2).

**Figure 3:**
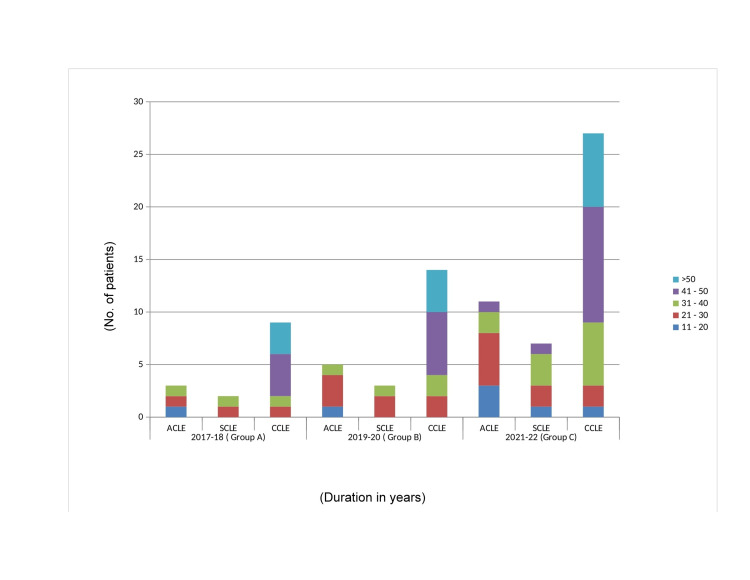
Age distribution. While CCLE is found more among the elderly age group, ACLE/SCLE is common in younger age groups. CLE, cutaneous lupus erythematosus; CCLE, chronic CLE; ACLE, acute CLE; SCLE, subacute SCLE

In the study groups (shown in Figure 3), CCLE was more common in the elder (41-50) age group; in contrast, acute CLE (ACLE) was more frequent in the relatively younger (11-30) age group. SCLE was more prevalent in the middle age group of 21-40 years.

**Figure 4:**
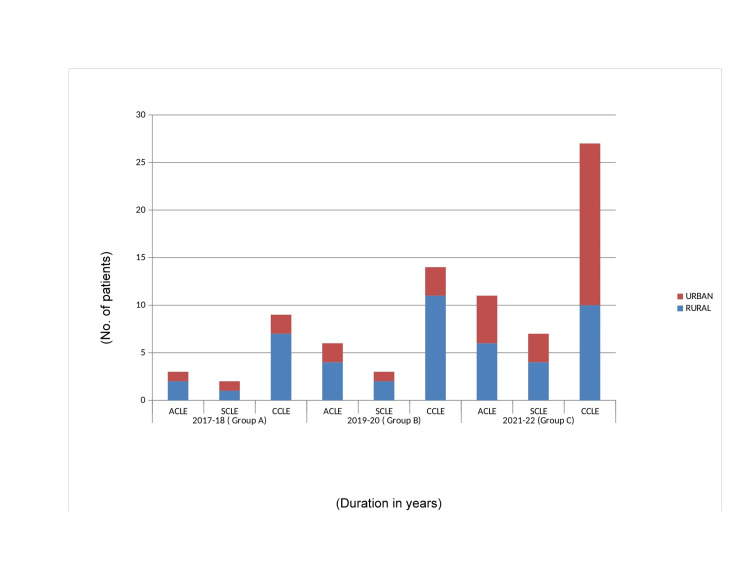
Area distribution: the figures indicate the habitat of the patients, i.e. rural/urban. An increased amount of urban people affected by CCLE in 2021-2022 is evident. CCLE, chronic CLE; CLE, cutaneous lupus erythematosus

The CLE was more prevalent among farmers and laborers. The majority of the patients resided in rural areas whereas more number of CCLE patients belonging to urban areas was observed in Group C (Figure 4).

**Figure 5:**
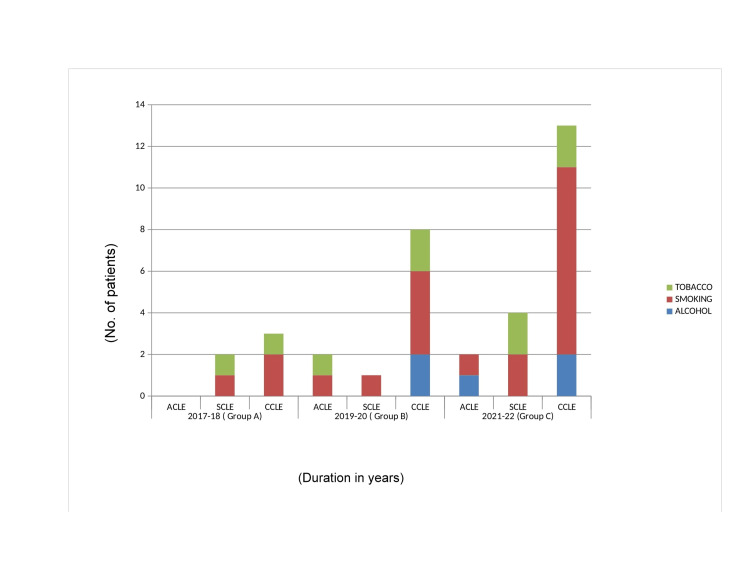
This figure describes the addiction patterns. Smoking and tobacco are the most common addictions.

Significant history of smoking was obtained especially in patients of CCLE than ACLE and SCLE as evident in Figure 5.

**Figure 6:**
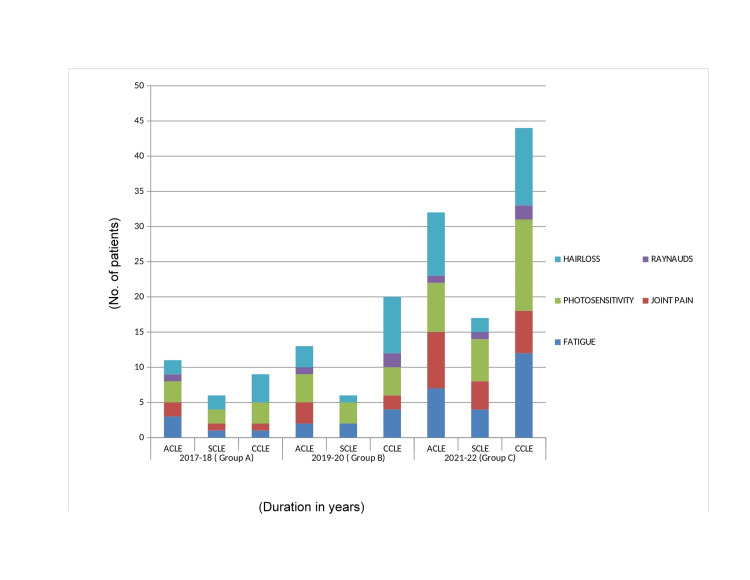
This figure shows different symptoms of CLE. Photosensitivity is the most common. CLE, cutaneous lupus erythematosus

Most of the patients presented with a variety of symptoms like hair loss, photosensitivity, fatigue, and arthralgia while photosensitivity was the most common as shown in Figure 6.

The most commonly associated systemic comorbidity was arthritis. Deranged hematological indices were the most common laboratory abnormality. The ANA antibodies were observed more frequently in patients with ACLE (90%) than in SCLE patients (69%). In contrast, 30% of the patients with CCLE were positive for ANA or extractable nuclear antigen (ENA) (22%). Table 2 shows the clinical profile of all 81 patients.

**Table 2:** The clinical profile of patients (N=81). *Drugs – Isoniazide, Minocycline, Terbinafine, Hydrochlorothiazide ACLE, acute CLE; SCLE, subacute CLE; CCLE, chronic CLE; CLE, cutaneous lupus erythematosus

Study period	2017-2018 (Group A)			2019-2020 (Group B)			2021-2022 (Group C)		
Cutaneous LE type	ACLE	SCLE	CCLE	ACLE	SCLE	CCLE	ACLE	SCLE	CCLE
Symptoms									
Fatigue	3	1	1	2	2	4	7	4	12
Joint pain	2	1	1	3	0	2	8	4	6
Photosensitivity	3	2	3	4	3	4	7	6	13
Raynaud’s phenomenon	1	0	0	1	0	2	1	1	2
Hair loss	2	2	4	3	1	8	9	2	11
Systemic comorbidities									
Arthritis	2	1	0	4	2	3	5	4	5
Nephritis	1	0	0	0	0	0	1	1	0
Serositis	2	0	0	2	0	0	4	0	0
Neurological	0	0	0	0	0	0	1	0	0
Addiction history									
Alcohol	0	0	0	0	0	2	1	0	2
Smoking	0	1	2	1	1	4	1	2	9
Tobacco	0	1	1	1	0	2	0	2	2
Family history of LE									
	1	0	0	1	0	0	4	1	0
H/O drugs*									
	1	0	0	0	1	0	2	1	0
H/O photodermatoses									
	2	1	5	4	2	8	7	3	18
ANA profile									
Anti-nuclear antibodies	3	1	3	5	2	4	9	6	8
Anti ena antibodies	2	0	3	2	1	3	8	5	5
Anti ssa antibodies	1	1	1	2	2	3	8	4	6
Anti ssb antibodies	0	1	1	0	2	4	2	4	3
Anti rnp antibodies	0	0	2	2	2	4	0	4	8
Anti sm antibodies	2	0	2	1	1	3	2	2	7
Anti ds-dna antibodies	2	0	3	2	1	4	3	2	8
Anti histone antibodies	1	0	0	1	0	1	2	1	0
Anti nucleosome antibodies	0	0	0	0	0	1	0	0	2

## Discussion

In the current study, data of eligible patients as per the data extraction sheet were collected for six consecutive years starting from 2017 to 2022. The collected data were then divided into three groups where each group consisted of patients visiting OPD over a period of 2 years. The study showed an increase in the number of LE cases rising about 157% in Group B than in Group A and an increase of 204% in Group C than in Group B. There was almost a 200% increase in ACLE, SCLE, and CCLE cases over each group. A steady increase of around 150%-300% was observed in female cases in each group while CCLE cases abruptly showed a 466% increase in males in Group C than in Group B. A 500% increase in urban population was observed in Group C over Group B while in the rural population, the rising trend showed constant changes.

This could be due to the migration of people from rural to urban areas for better socio-economic conditions. UV light exposure, which is known to be a triggering factor for LE might be playing a greater role in the development of this disease in farmers and laborers who work under direct sunlight [5-6]. Moreover, there is also a change in food intake habits especially containing fewer antioxidants and various substance abuse commonly observed in the urban population. However, genetic susceptibility, autoimmune induction, immune system changes, drugs, and some infections are the main causes of LE [7]. Among these, some viral infections, and self-medication of drugs during the COVID pandemic like proton pump inhibitors, antibiotics, and NSAIDs could have contributed to the rise of cases in Group B and Group C also [8-12].

In our study female predominance was noted with male to female ratio of 1: 2. This is consistently demonstrated by many studies including those conducted by Rees et al. (2016) and Mowla et al. (2022) [13-14]. In general, the percentage of female patients ranges from 78% to 96% in most studies, with a female-to-male ratio of even up to 10: 1 [15-16]. This is especially observed in the 15- to 64-year age group, where ratios of age and sex-specific incidence rates show a 6- to 10-fold rise in female patients. Hormonal changes are attributed to these age-related differences in the female-male ratios. But the greater number of males presented to us with CCLE in 2021-22 in contrast to many studies including that of Bajaj et al. (2010) and Hong et al. (2019) [17-18]. The rationale behind such an observation may be attributed to more number of previously migrant male laborers coming back to our city for many ongoing infrastructure projects abandoning their work in other states after the COVID period. As we know CLE patients are highly photosensitive; therefore, disease prevalence could be higher when people work under direct sunlight.

In the current study mean age was 37.65 ± 13.7 years (range 11-66 years) similar to Mowla et al. (2022) where the mean age was 30.0 ± 11.7, ranging between 11 and 65 years [14]. As it is noteworthy that acute cutaneous LE typically presents in the third decade of life, subacute cutaneous lupus erythematosus (SCLE) occurs primarily in young to middle-aged women and CCLE occurs more frequently in women in their fourth and fifth decade of life as suggested by Ng et al. (2000) and Fabbri et al. (2003), our results were no different [19-20]. While most of the LE patients were found to be residing in rural areas as observed in studies conducted by Mowla et al. (2022) and Williams et al. (2021) [14, 21]. CCLE was more prevalent in farmers and laborers possibly due to working under the outdoor sun. This could have triggered the development of the disease. History of smoking was found to be associated with higher disease activity, low response to drugs, and increased risk for systemic organ involvement in studies conducted by Turchin et al. (2009) and Piette et al. (2012), while our observations yielded quite similar results [22-23]. The ANA antibodies were observed more frequently in patients with ACLE (89%) than in SCLE patients (75%). In contrast, 30% of the patients with CCLE were positive for ANA while other studies by Tebbe et al. (1997) and Okon et al. (2013) observed that positive ANA is found in 95% of ACLE patients, 70%-80% of SCLE patients, and a lower incidence among CCLE patients [24-25].

Limitation

This is a pilot study of CLE with its various aspects in our area. This was a single-center study with a limited sample size. Due to its chronicity and high recurrence rate, longer study periods with regular follow-ups are required to obtain established changes in trends.

## Conclusions

The current study noticed an increasing trend of all types of CLE among patients reporting to a tertiary care center in Odisha. Among the reported patients two third were females and around three-fifth were from rural areas. While photosensitivity was the most common symptom, arthritis was the major systemic co-morbidity observed. The rising trend in LE should be investigated for other environmental factors like climatic changes, lifestyle, behavioral changes in addition to viral infection, and drug factors. These types of studies should be carried out in other similar types of geographical areas and conditions to strengthen our findings. The current study findings could be helpful for public awareness, prevention of morbidity, and healthy lifestyle.
